# Case of Crancum Oris, with Necrosis of a Large Portion of the Inferior Maxillary Bone, Followed by Recovery

**Published:** 1853-01

**Authors:** T. Herbert Barker

**Affiliations:** London.


### ARTICLE XVI.

*Case of Cancrum Oris, with Necrosis of a large portion of the Inferior Maxillary Bone, followed by Recovery.* By T. HERBERT BARKER, M. D., F. R. C. S., London.

JOSIAH HILL, of Cope, near Bedford, aged five years and a quarter, was recovering from typhus, which had attacked several members of the family, when a swelling of the right cheek made its appearance, rapidly increasing, and becoming red, hard, and shining. The swelling was the most prominent and tense in the lower port of the cheek, opposite the lower jaw; and the surrounding œdema extended to the nose, right eyelids, (which were closed,) and to the right ear. On examining the interior of the mouth, which was done with some difficulty, owing to the swelling, irregular and jagged ulceration of the cheek and gum was observed, at a point corresponding with the central hardness, and covered with a dark-colored shreddy slough. On the third day a bright red spot appeared externally in the centre of the swelling, which speedily assumed a purplish hue, and the cuticle became raised by a sanious fluid underneath. At this period there was considerable fetor of the breath, and a flow of offensive saliva from the mouth. The skin was hot, the pulse quick, thirst urgent, and the patient was delirious at night. The central livid spot increased rapidly in size, and on the sixth day was larger than a crown piece, circular, and with a deep red color of the surface surrounding it. On the seventh day a line of demarcation could be detected, and a small quantity of fetid discharge escaped from the slough, which gradually began to separate at its edges. The saliva, which had hitherto been pale, now became of a dirty-brown color, and extremely fetid, so that the room was scarcely tolerable. The slough gradually loosened, and came away on the sixteenth day, when a large surface of the inferior maxillary bone was found to be denuded, and of a brownish color. The surrounding œdema gradually subsided, and the febrile symptoms gave way to those of extreme exhaustion. The large opening through the cheek began to contract in a few days, and the edges to look cleaner, and to be covered with healthy granulations. An offensive discharge continued to flow from the mouth, as well as from the opening in the cheek. In five weeks from the commencement of the swelling, the opening through the cheek had contracted to the size of a shilling, through which the denuded bone was still visible. Soon after this period the patient directed my attention to a jagged edge of bone within the mouth, which appeared just to the right of the symphysis of the lower jaw-bone. The right half of the inferior maxillary bone appeared to be somewhat raised above its natural level, but was not very movable. This jagged edge of bone became more prominent, and somewhat looser, from day to day; it caused some inconvenience to the tongue, prevented him from closing his mouth, and it was with some difficulty that he could be fed with a teaspoon. In eight weaks from the commencement of the disease it was sufficiently loose to enable me to extract the entire piece of bone. After the separation of the bone, the opening through the cheek gradually contracted in size, and cicatrized, leaving a deep depression, capable of admitting the end of a finger. The necrosed portion of bone is nearly two inches in length, and consists of so much of the body of the inferior maxillary bone as contained five of the temporary teeth on the right side. Two of the permanent teeth, (the canine and first molar,) may be observed, just projecting into the sockets of the temporary teeth. The necrosed portion separated from the living bone anteriorly, just external to the central incisor tooth, and posteriorly immediately behind the last temporary molar tooth, and in front of the ramus of the jaw.

The treatment pursued was the assiduous application of warm fomentations in the first instance, and, on the appearence of the gangrenous spot, of carrot poultices. After the separation of the slough, weak sulphate of zinc lotion was kept applied, by means of lint, covered with oiled-silk, and the edges were touched from time to time with the nitrate of silver. Quinine, and afterwards the carbonate of ammonia, were administered internally, and a liberal supply of strong beef-tea, mutton, broth and wine, was allowed.

The present appearances of the patient, (five years after the separation of the bone,) are the following:

*Externally*, a deep cicatrized depression is observed, the site of the original slough; the angle of the jaw on the right side is observed to be an inch and a half nearer the symphysis than on the left side, which gives that side of the face a somewhat contracted and oblique appearance.

*Internally*, on the right side, only one tooth is observed—namely, the central incisor, which is somewhat below the level of the incisor of the left side; the space between this and the ramus of the jaw is occupied with a narrow line of new bone, which has been thrown out between the two points, and covered with a mucous surface. Mastication is performed efficiently on the left side.

*Remarks.*—This case corresponds with the second variety of cancrum oris described by Dr. Cuming in the fourth volume of the "Dublin Hospital Reports," and is interesting in consequence of the extent of the disease, and of the necrosis and separation of bone, followed by recovery. Dr. Cuming says, that in every instance that he had met with, this disease had occurred when the constitution had been debilitated by the existence of previous and protracted disease. Dr. Marshall Hall also states, in the fifteenth volume of the *Edinburgh Medical and Surgical Journal*, that in all the cases which had come to his knowledge, it had been preceded by fever, acute disorder of the digestive organs, typhus, inflammation of the lungs, variola, rubeola, or scarlatina, and regards it as a consequence of the exhaustion, debility, or irritation produced by previous disease.

In three other cases which have came under my notice, two occurred after typhus, and one as a sequela of scarlet fever. In every case the mucous membrane was the first attacked, the sloughy ulceration within the mouth having been observed before the external gangrenous spot made its appearance, thus confirming the views of Dr. Cuming, and of MM. Rilliet and Barthez, Baron and Dr. West, referred to in the excellent "Lectures on the Diseases of Children" by the last author.

All the cases occurred at or about the same age; and two of them terminated fatally with low fever, diarrhea, and exhaustion, before any line of separation of the gangrenous portion had formed. The other case of recovery was one in which the upper jaw was involved in the disease; and a portion of bone, containing a primary molar tooth, came away through the ulcer in the cheek, leaving a permanent and unsightly cicatrized depression.

In consequence of some resemblance of these cases to the ulceration and inflammation of the mouth produced by mercury, they have frequently been attributed to the administration of that drug; but from the number of cases which have occurred where mercury had not been given, there can be no doubt that there is not necessarily any connection between the two, and certainly this remedy had not been used in any form in the cases under my notice. It may be remarked, that cancrum oris is confined to one side of the face, whereas, from the use of mercury, the gums of both jaws are affected, as well as on both sides. The fetor, too, produced by mercury, is very different from the highly-offensive and putrid odor of cancrum oris—a difference readily recognized by any one who has noticed the fetor from both causes.

With regard to the treatment of cancrum oris I have nothing more to state, than that I am inclined rather to have recourse to fomentations, poultices, and the nitrate of silver, than to the strong acids which have been recommended by some practitioners. Internally the cordial and tonic plan of treatment is clearly indicated.—*Provincial Med. & Surg. Journal.*

